# Handling, infectious agents and physiological condition influence survival and post-release behaviour in migratory adult coho salmon after experimental displacement

**DOI:** 10.1093/conphys/coaa033

**Published:** 2020-05-18

**Authors:** J M Chapman, A K Teffer, A L Bass, S G Hinch, D A Patterson, K M Miller, S J Cooke

**Affiliations:** 1 Fish Ecology and Conservation Physiology Laboratory, Department of Biology, Carleton University, 1125 Colonel By Drive, Ottawa, ON, K1S 5B6 Canada; 2 Pacific Salmon Ecology Laboratory, Forest and Conservation Sciences, University of British Columbia, Vancouver, BC, Canada. Salmon Ecology and Conservation Laboratory, Department of Forest and Conservation Sciences, University of British Columbia, Vancouver, BC V6T 1Z4, Canada; 3 Cooperative Resource Management Institute, School of Resource and Environmental Management, Fisheries and Oceans Canada, Burnaby, BC, Canada. Fisheries and Oceans Canada, Cooperative Resource Management Institute, School of Resource and Environmental Management, Simon Fraser University, Burnaby, BC V5A 1S6, Canada; 4 Fisheries and Oceans Canada, Molecular Genetics Section, Pacific Biological Station, Nanaimo, BC V9T 6N7, Canada

**Keywords:** telemetry, wildlife disease, gene expression, fisheries, migration, pathogen

## Abstract

For Pacific salmon captured and released by fisheries, post-release behaviour and survival may be influenced by their health and condition at time of capture. We sought to characterize the interactions between infectious agent burden, fish immune and stress physiology and fisheries stressors to investigate the potential for capture-mediated pathogen-induced mortality in adult coho salmon *Oncorhynchus kisutch.* We used radio-telemetry paired with high-throughput qPCR of non-lethal gill biopsies for infectious agents and host biomarkers from 200 tagged fish experimentally displaced and exposed to various experimental fisheries treatments (gill net entanglement, recreational angling and recreational angling with air exposure vs. non-sampled control). We characterized relationships among post-release behaviour and survival, infectious agent presence and loads, physiological parameters and transcription profiles of stress and immune genes. All infectious agents detected were endemic and in loads consistent with previous adult Pacific salmon monitoring. Individuals exposed to fisheries treatments were less likely to reach spawning habitat compared to controls, and handling duration independent of fisheries gear had a negative effect on survival. High infectious agent burden was associated with accelerated migration initiation post-release, revealing behavioural plasticity in response to deteriorating condition in this semelparous species. Prevalence and load of infectious agents increased post-migration as well as transcription signatures reflected changes in immune and stress profiles consistent with senescence. Results from this study further our understanding of factors associated with fisheries that increase risk of post-release mortality and characterize some physiological mechanisms that underpin migratory behaviour.

## Introduction

Associations among pathogens and their hosts in wildlife populations are relevant to conservation efforts but remain relatively under-studied due to logistical constraints (Grenfell *et al*. 1995). For cryptic aquatic species, the majority of information available on host–pathogen relationships is derived from monitoring of disease that occurs in domesticated aquaculture strains in captive facilities (Kurath and Winton 2011). Pathogen-related disease in wild aquatic animals is generally only considered a conservation issue when observable mortality or large-scale population declines occur during cases of epizootic outbreaks (e.g. viral hemorrhagic septicemia in the Laurentian Great Lakes). As a result, surveys of infective agents in aquatic wildlife populations generally investigate relatively rare events or anomalies and do not assess infectious agents in the context of general ecology and conservation (Miller *et al*. 2014). Research has demonstrated that microbial infectious agents and their effect on fish are a complex relationship that depends on multiple factors including but not limited to environmental conditions, host life-history stage, immunocompetency and co-infection dynamics (Tort 2011). As aquatic ecosystems continue to experience change as a result of human activities (Dudgeon *et al*. 2006; Reid *et al.* 2019), it is increasingly necessary to characterize pathogen dynamics outside of aquaculture and episodic disease events to focus instead within the context of ‘natural’ pathogen dynamics and impacts of anthropogenic stressors. As infectious disease is often believed to be the ultimate cause of mortality in stressful environments (Joseph *et al*. 2013; Miller *et al*. 2014) and stressors rarely occur in isolation outside of laboratory settings, it is necessary to investigate synergistic effects of multiple stressors when considering stress in wild organisms (Crain *et al*. 2008).

While many anthropogenic stressors are broad-scale with obvious negative connotations, such as climate change or habitat destruction, fish can also experience acute stress as a result of conservation actions such as release from fisheries. Fisheries interactions represent a multifactorial stressor that can impose exhaustive exercise, physical injury and hypoxia upon captured fish (Davis 2002; Patterson *et al*. 2017). Stressors by definition induce stress responses at primary, secondary and tertiary organismal scales (Barton 2002), which can trigger behavioural changes and a cascade of alterations in physiological processes. These can include gene expression and protein synthesis, metabolism and energetics and immune and endocrine function (Tort 2011). The link between stress and fish immunocompetency has been demonstrated to be particularly complex; depending on the type of stressor and recipient’s condition at time of induction, a cellular stress response can both activate (Aluru and Vijayan 2009; Kassahn *et al*. 2009) and suppress (Dhabhar 2002; Van Rijn and Reina 2010) immune-associated genes. As individual genes are generally involved in multiple physiological pathways and may respond to multiple stressor types, detection of a specific stressor based on shifts in expression of single gene transcripts will likely yield erroneous conclusions (Houde *et al*. 2019). Alternately, assessing patterns of transcription for a broad range of immune and stress-related genes may help characterize observed outcomes after exposure to acute stressors. We have demonstrated that specific stressor and disease states can be accurately discriminated based on curated biomarker (gene) panels that are specifically co-expressed under specific stressor conditions (Miller *et al*. 2017; Akbarzadeh *et al*. 2018; Houde *et al*. 2019), and these panels can be effective even in multistressor scenarios (Houde *et al*. 2019). Moreover, tracking and holding studies have revealed transcriptional signatures associated with shifts in migratory behaviour, survival, age and/or environmental exposure that have been demonstrated across multiple studies (e.g. ‘mortality-related signature’ or MRS described in Miller *et al*. 2011, Drenner *et. al* 2018, Stevenson *et al*. 2019; viral disease response or ‘VDD’ in Jeffries *et al.* 2012; Miller *et al*. 2017; Twardek *et al*. 2019; Stevenson *et al*. 2019).

Although the goal of many fisheries is to capture and harvest fish, many fish are also released (i.e. capture-and-release) to comply with regulations or voluntarily due to conservation ethic (e.g. recreational voluntary catch-and-release) or lack of market in the case of commercial fisheries. Fisheries stressors include exhaustive exercise, air exposure and physical injury from contact with fishing gear (hooks, nets) and handling (reviewed in Raby *et al.* 2015b). These interactions can leave released fish physiologically compromised (reviewed in Davis 2002; Wilson *et al*. 2014) and potentially vulnerable to pathogen infection and disease progression (Patterson *et al*. 2017). How a capture-and-release interaction may influence disease-related mortality is termed ‘capture-mediated-pathogen-induced mortality’, and while estimates of post-release mortality in general have been determined for many capture-and-release fisheries, the level of pathogen-induced mortality that occurs in the wild is unknown.

Biotelemetry allows researchers to track the migration behaviour and post-release survival and reproductive success of fish released from fisheries (Donaldson *et al*. 2008), such as Pacific salmon *Oncorhynchus* spp. (Donaldson *et al*. 2012; Raby *et al*. 2012). By combining physiological sampling with telemetry, detailed questions regarding host condition at time of release can be addressed (reviewed in Cooke *et al*. 2013; Patterson *et al*. 2016). This approach is particularly powerful when combined with advanced pathogen screening, allowing the empirical characterization of infection and physiological state at time of tagging to be directly related to mortality outcomes (Miller *et al*. 2014; Bass *et al*. 2017). Further, simultaneously assessing host transcription profiles of immune and stress-related genes creates a comprehensive picture of not only the microparasite community present within the host at the time of tagging but also whether the host exhibits physiological or transcriptional responses associated with infections (e.g. Teffer *et al*. 2017, 2019). This methodology allows for the investigation of fundamental research questions regarding host–pathogen interactions and factors that influence these relationships, while also addressing population-scale effects potentially important for the management and conservation of host species. For Pacific salmon, this technique has been used to identify new and endemic pathogens in British Columbia’s adult (Bass *et al*. 2017) and juvenile salmon (Tucker *et al*. 2018) and to identify pathogenic microbes that influence migratory survival of juvenile salmon (Jeffries *et al.* 2012) and demonstrate links between host transcriptional responses and survival in both smolts and migratory adults (Miller *et al*. 2011, 2014, 2017; Jeffries *et al.* 2012; Teffer *et al*. 2018).

To understand how infectious agent dynamics and fish physiology and immune responses influence post-release impairment and migration success, we measured infectious agent loads and host physiological and transcriptional status in adult coho *O. kisutch* exposed to standardized fishery treatments. Adult coho salmon that have entered fresh water are vulnerable to recreational and commercial in-river fisheries that use a variety of gear types, including hook and line and gillnets. Coho salmon populations are generally regarded as being in decline in the Fraser River basin with one population categorized as Threatened by the Committee on the Status of Endangered Wildlife in Canada (Interior Fraser River coho salmon; COSEWIC 2016). Releasing coho salmon following capture is a tactic that is used in Fraser River commercial fisheries (when coho salmon are captured as by catch) and recreational fisheries (depending on region and stock origin e.g. hatchery versus wild) to ensure that fisheries activities do not further exacerbate declines in adult coho escapement. Yet, there have been very few studies on the effects of releasing coho salmon from capture on their subsequent migration survival (e.g. Raby *et al*. 2012, 2015a) and none that have examined the effects of individual condition and infectious agent burdens on migration success. Recent experimental holding studies on adult coho salmon demonstrated links between fisheries stressors and transcriptional profiles, infectious agent communities, and survival (Teffer *et al*. 2019), but such links have not been identified the wild. To investigate these factors, we exposed migrating adult coho salmon to experimental fisheries simulations, measured infection burdens and host transcriptional responses in gill, quantified stress indices in blood and then used radio telemetry to relate post-release migratory behaviour and survival to upstream spawning habitat to physiological and disease-associated metrics. To determine changes in infectious agent burdens and host condition, fish were opportunistically recaptured post-migration and experimentally displaced 75 km down river for experimental treatments, biopsy and tagging. We tested the hypothesis that the severity of fisheries treatment, infection burden and physiological status at release influence migration rate and survival and that infection burden would increase in fish recaptured post-migration (as observed in other Pacific salmon examined during spawning migrations (e.g. Bass *et al*. 2019)). We predicted that fisheries treatments and high infection burden would be related to migratory impairment and decreased survival and that infectious agent diversity and relative load increased during migration.

## Methods

### Capture and transport

**Figure 1:**
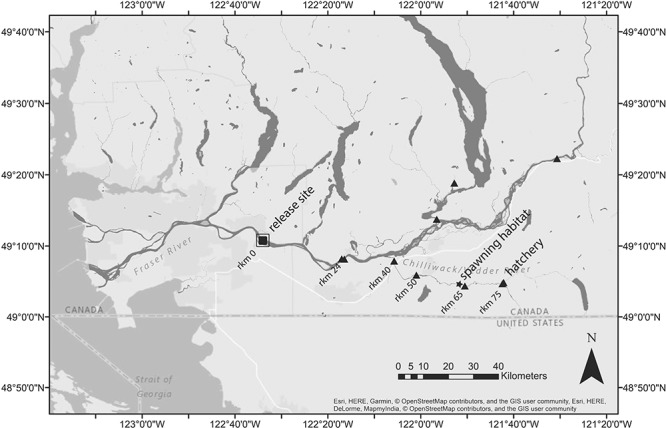
Map of the study area, including release site, receiver locations (triangles) and the beginning of spawning habitat for Coho salmon experimentally displaced from the Chilliwack River Hatchery and exposed to experimental fisheries treatment prior to tagging, sampling and release

Adult coho salmon were collected at the Fisheries and Oceans Canada Chilliwack River Hatchery in Chilliwack, British Columbia (20–24 October 2014, Fig. 1). Individuals present at the hatchery at the time of sampling had already migrated from the ocean to the hatchery; however, work began early in the migration period of this population to ensure coho salmon, which tend to enter rivers early and stage near spawning grounds for weeks prior to spawning (McPhail and Lindsey 1970), were in good condition with several weeks remaining before spawning commenced. We chose to use the hatchery as a collection site rather than attempting to intercept fish during migration to avoid stress and injury associated with in-river capture methods, to condense the number of days across which fish were released and to ensure robust sample sizes. At time of capture, all fish were silver in colour with no secondary sexual characteristics indicating gonadal maturation is not complete and relatively recent river entry (Groot and Margolis 1991). Two hundred adult coho salmon were dip-netted from hatchery collection channels and immediately transferred to an aerated truck-mounted transport tank filled with sand-filtered, UV-treated water sourced from within the same watershed (10–12°C). Selection of fish was done haphazardly. Water was oxygenated using compression canisters, and temperature and dissolved oxygen were monitored continuously during transport. Fish were then transported 75 river kilometres downstream (~1 h transit time; Fig. 1) and transferred using dip nets to in-river holding pens with front to back flow-through. All fish survived capture and transportation. Fish were monitored during holding and allowed to recover for 1 h prior to experimental treatments. All research complied with Canadian Council for Animal Care Protocol #102022 and was conducted under Movement of Live Fish in British Columbia permit # 13533 and Scientific License XR 3622014.

#### Experimental treatments

Experimental treatments aimed to replicate stress and exertion associated with in-river fisheries; our team has worked alongside fishers in the study region to design treatments that simulate real-world aspects of fisheries interactions commonly experienced by migrating salmon. Treatments were randomly assigned and included two recreational angling simulations representing best practices (no air exposure, biopsied) and poor practices (1 min air exposure, biopsied), gill-net by-catch simulation (1 min air exposure, biopsied) and a non-treated control (no biopsy). Air exposure treatments were included for angling and gill net treatments to represent the typical experience for these fisheries (Cook *et al*. 2019), and a no air exposure angling treatment was included to assess if current ‘best handling practices’ may potential mitigate the deleterious effects of air exposure (Brownscombe *et al*. 2017). Experimental treatments were conducted in a large (500 L) tank supplied with continually pumped river water. For recreational angling simulations, fish were transferred to the measuring trough and manually hooked through the maxilla using a no. 2 circle hook rigged on a 4.5-kg test monofilament fishing line and released into the experimental arena for 2 min. Tension was placed on the line by the experimental angler to encourage the fish to fight as it would in a typical angling event. Fish were then either immediately submerged in a tagging trough with a continual flow of fresh river water over the gills and body (simulating best angling practices via fast return to the water) or exposed to 1 min of air exposure prior to entering the sampling trough (simulating poor handling practices). Hook removal occurred either during the air exposure treatment, or immediately after transfer to the tagging trough while submerged.

Gillnet simulations consisted of a 3-min experimental entanglement in 140 mm mesh commercial regulation gillnet. Netting was held in an aluminium frame at low tension such that mesh was open but not taught to simulate suspended netting potentially encountered in the river by migrating fish. Each fish was individually dip-netted from the in-river holding pen, transferred to a corner of the experimental arena and released to swim into the gillnet. After 3 min of entanglement, fish were removed and treated with 1 min of air exposure as described above and then transferred to the tagging trough for biopsy and tagging. In all cases, total handling time was recorded for each fish from the time it was dip-netted out of the holding tank to when it was released.

#### Biopsy and tagging procedure

The tagging trough was foam-lined with flow-through water pumped continually from the river. Fish were measured (fork length), biopsied for gill tissue (two filament tips, ~ 2 mm in length) and blood (2 mL from the caudal vasculature; 21-gauge needle and heparinized 3-mL Vacutainer, Becton-Dickson, NJ; as per Miller *et al*. 2014; Bass *et al*. 2017). Gill tissue was immediately transferred into 2 mL RNAlater® for RNA stabilization and preservation for transcript analysis. Blood samples were kept in an ice slurry for up to 15 min and then centrifuged for 6 min at 7000 *g* to isolate plasma, which was transferred (~1.5 mL) to microtubes and flash-frozen in liquid nitrogen for subsequent analysis of metabolites and hormones. No biopsy was taken from fish in the control group (no treatment and no biopsy; *n* = 50) prior to tagging. All fish were internally tagged by gastric insertion with Sigma Eight Inc. (Markham, ON) coded radio tags programmed for a 4-s signal transmission interval and externally tagged with individual ID spaghetti tags (Floyd Manufacturing, Seattle, WA), then released. Signal transmission and ID decoding were confirmed immediately prior to deployment using a Lotek Wireless (Newmarket, ON) SRX-600 radio telemetry receiver.

Nine fixed Sigma-8 Orion radio receivers were installed upstream of the release site: seven along the predicted migration route, and three up-stream of the Chilliwack–Vedder confluence with the Fraser River (Fig. 1). Manual tracking of the system occurred every 48 h along roadways adjacent to the river system where possible and by foot at river access points. Migration progress was estimated based on sequential detection at receiver stations up-river of the release site and by manual tracking (Lotek SRX 600). Migration ‘success’ was defined as detection of fish at or beyond river km 65, where habitat suitable for spawning begins and spawning has been observed (Chilliwack River Hatchery staff, personal comm.). Fish that were detected upstream of the release site but not detected at or beyond river km 65 were considered en-route mortalities, while fish that were not detected after release are considered immediate post-release mortalities.

Lethal sampling was conducted at the collection location (Chilliwack River Hatchery) to provide baseline condition, gene expression, blood physiology and microbial infection data at the beginning and end of the study. Any tagged fish that was recaptured upon return to the Chilliwack Hatchery was also lethally sampled. On the first and last days of the study, *N* = 10 adult coho salmon were taken directly from the hatchery channel and immediately euthanized by cerebral percussion (referred to as baseline initial and baseline final respectively). Length, weight, blood and tissue samples and reproductive status were collected for each fish. For comparative purposes, this study uses biomarker and pathogen data isolated from gill tissue only. Gill biopsies from sacrificed fish were taken from a similar location on the gill and in a similar manner and size to nonlethal biopsies.

#### Blood assays and molecular techniques

Plasma samples were processed at the Department of Fisheries and Oceans Canada (DFO) West Vancouver Laboratory, West Vancouver, BC. Plasma sodium, potassium and chloride were measured to investigate potential osmoregulatory disturbance (Barton 2002) with a Cole Parmer single-channel flame photometer (model 2655–00), and glucose and lactate were measured to assess the metabolic response to stress with a YSI STAT Plus glucose/lactate analyzer (model 2300). All analyses were run in duplicate and averaged following procedures outlined in Farrell *et al*. (2001). Plasma testosterone, estradiol and cortisol concentrations were processed and run in duplicate and measured as per manufacturer’s instructions using ELISA kits (Neogen Corp., Lansing, MI, USA). Testosterone and estradiol levels for blood-sampled tagged fish were compared to those from baseline destructives samples (hatchery) where sex was known (*n* = 40) to inform sex assignment of tagged fish. Consequently, sex is unknown for control fish as no blood was taken.

Gene expression of host genes and infectious agent prevalence and relative loads were examined in gill tissue using high-throughput qPCR on the Fluidigm BioMark Dynamic Array™ Gene Expression platform at the DFO Molecular Genetics Laboratory in the Pacific Biological Station, Nanaimo, BC (Tables 1 and 2). Biomarkers believed or known to be linked with infection and mortality were selected. To determine the presence of infectious agents within samples, a pool of all samples was screened for 46 infectious agents expected or known to cause disease in salmon globally (a full list of screened pathogens is available in Supplemental Material). Only those that were positive in the pooled sample were run on individual samples. TaqMan™ assays used in screening were designed to target microbe RNA to allow identification of RNA viruses and microparasites in active states. Consequently, qPCR quantification represents microbial productivity rather than absolute quantification, described hereafter as relative load. Details on sensitivity and specificity of each microbe assay, sequence sources and repeatability of the following RNA extraction, normalization, targeted amplification and final HT-RT-qPCR using this platform are outlined in the Canadian Science Advisory Secretariat validation of the BioMark for infectious agent monitoring in salmonids (Miller *et al*. 2016).

**Table 1:** Abbreviations, names and groups of infectious agents detected in migratory adult Coho salmon collected in the Chilliwack River system using high-throughput qPCR

Assay ID	Infectious agent	Group	LOD (40-Ct)	Mean relative load ± SD	TaqMan primer sequences (5′–3′) Probe sequence (FAM-5′–3’-MGB)	Source sequence
ae_sal	*Aeromonas salmonicida*	Bacterium	14.4	12.2	F - TAAAGCACTGTCTGTTACC	Keeling *et al*. 2013
					R - GCTACTTCACCCTGATTGG	
					P - ACATCAGCAGGCTTCAGAGTCACTG	
c_b_cys	*Candidatus* Branchiomonas *cysticola*	Bacterium	14.3	27.4 ± 3.1	F - AATACATCGGAACGTGTCTAGTG	Mitchell *et al*. 2013
					R - GCCATCAGCCGCTCATGTG	
					P - CTCGGTCCCAGGCTTTCCTCTCCCA	
fl_psy	*Flavobacterium psychrophilum*	Bacterium	10.5	17.8 ± 2.8	F - GATCCTTATTCTCACAGTACCGTCAA	Duesund *et al*. 2010
					R - TGTAAACTGCTTTTGCACAGGAA	
					P - AAACACTCGGTCGTGACC	
rlo	Rickettsia-like organism	Bacterium	14.8	22.5	F - GGCTCAACCCAAGAACTGCTT	Lloyd *et al*. 2011
					R - GTGCAACAGCGTCAGTGACT	
					P - CCCAGATAACCGCCTTCGCCTCCG	
env	Erythrocytic necrosis virus	Virus	15.1	16.4	F - CGTAGGGCCCCAATAGTTTCT	Purcell *et al*. 2013
					R - GGAGGAAATGCAGACAAGATTTG	
					P - TCTTGCCGTTATTTCCAGCACCCG	
cr_sal	*Cryptobia salmositica*	Parasite	15.7	13.6 ± 1.1	F - TCAGTGCCTTTCAGGACATC	Miller *et al*. 2016
					R - GAGGCATCCACTCCAATAGAC	
					P - AGGAGGACATGGCAGCCTTTGTAT	
ce_sha	*Ceratonova shasta*	Myxozoan	11.5	18.1 ± 6.7	F - CCAGCTTGAGATTAGCTCGGTAA	Hallett and Bartholomew 2006
	(formerly *Ceratomyxa shasta*)				R - CCCCGGAACCCGAAAG	
					P - CGAGCCAAGTTGGTCTCTCCGTGAAAAC	
de_sal	*Dermocystidium salmonis*	Parasite	14.5	18.4 ± 1.9	F - CAGCCAATCCTTTCGCTTCT	Miller *et al*. 2016
					R - GACGGACGCACACCACAGT	
					P - AAGCGGCGTGTGCC	
ic_mul	*Ichthyophthirius multifiliis*	Parasite	16.3	16.2 ± 6.4	F - AAATGGGCATACGTTTGCAAA	Miller *et al*. 2016
					R - AACCTGCCTGAAACACTCTAATTTTT	
					P - ACTCGGCCTTCACTGGTTCGACTTGG	
lo_sal	*Loma salmonae*	Parasite	14.6	22.8 ± 8.2	F - GGAGTCGCAGCGAAGATAGC	Miller *et al*. 2016
					R - CTTTTCCTCCCTTTACTCATATGCTT	
					P - TGCCTGAAATCACGAGAGTGAGACTACCC	
pa_ther	*Paranucleospora theridion*	Parasite	11.8	17.8 ± 3.7	F - CGGACAGGGAGCATGGTATAG	Nylund *et al.* 2010
					R - GGTCCAGGTTGGGTCTTGAG	
					P - TTGGCGAAGAATGAAA	
pa_pse	*Parvicapsula pseudobranchicola*	Myxozoan	14.8	13.6 ± 7.5	F - CAGCTCCAGTAGTGTATTTCA	Jørgensen *et al*. 2011
					R - TTGAGCACTCTGCTTTATTCAA	
					P - CGTATTGCTGTCTTTGACATGCAGT	
pa_min	*Parvicapsula minibicornis*	Myxozoan	10.4	18.5 ± 5.2	F - AATAGTTGTTTGTCGTGCACTCTGT	Hallett and Bartholomew 2009
					R - CCGATAGGCTATCCAGTACCTAGTAAG	
					P - TGTCCACCTAGTAAGGC	
sp_des	*Sphaerothecum destruens*	Parasite	13.5	12.5 ± 6.1	F - GCCGCGAGGTGTTTGC	Miller *et al*. 2016
					R - CTCGACGCACACTCAATTAAGC	
					P - CGAGGGTATCCTTCCTCTCGAAATTGGC	

LOD = limit of detection

**Table 2:** Abbreviations, gene names, functional groups and primer/probe sequences for biomarkers associated with stress and immune function and reference genes assessed in migratory adult Coho salmon using high-throughput qPCR

**Assay name**	**Gene name**	**Functional group**	**Primer and probe sequences**	**Efficiency**	**Source**
ATP5G3-C	ATP synthase lipid-binding protein	Stress	F - GGAACGCCACCATGAGACA	0.975	Workenhe *et al*. 2009
			R - CGCCATCCTGGGCTTTG		
			P - AGCCCCATTGCCTC		
CD83_onmy	Complement factor CD83	Adaptive immunity	F - GATGCACCCCTTGAGAAGAA	1	Raida *et al*. 2011
			R - GAACCCTGTCTCGACCAGTT	
			P - AATGTTGATTTACACTCTGGGGCCA	
IL-1R_onmy1	Interleukin 1 receptor	Innate immunity	F - ATCATCCTGTCAGCCCAGAG	0.975	Raida *et al*. 2011
			R - TCTGGTGCAGTGGTAACTGG	
			P - TGCATCCCCTCTACACCCCAAA	
JUN	Transcription factor	Osmoregulation/stress	F - TTGTTGCTGGTGAGAAAACTCAGT	0.905	In house
			R - CCTGTTGCCCTATGAATTGTCTAGT	
			P - AGACTTGGGCTATTTAC	
CXCR4	C–X–C motif chemokine receptor 4	Immune	F - GGAGATCACATTGAGCAACATCA	0.93	In house
			R - GCTGCTGGCTGCCATACTG		
			P - TCCACGAAGATCCCCA		
FKBP	FK-binding protein	Immune	F - GCACGCCGGACTTTGC	1.1	In house
			R - GAGTTGGGTGGGATGATACCA	
			P - TATGGCAGCAAAGGG	
HSP90a	Heat shock protein 90-alpha (alike)	Stress	F - TTGGATGACCCTCAGACACACT	0.97	In house
			R - CGTCAATACCCAGGCCTAGCT	
			P - CCGAATCTACCGGATGAT	
IgMs_onmy	Immunoglobulin	Adaptive immunity	F - CTTGGCTTGTTGACGATGAG	1.09	Raida *et al*. 2011
			R - GGCTAGTGGTGTTGAATTGG	
			P - TGGAGAGAACGAGCAGTTCAGCA	
IL-11	Interleukin 11	Innate immunity	F - GCAATCTCTTGCCTCCACTC	1.01	Raida and Buchmann 2008
			R - TTGTCACGTGCTCCAGTTTC	
			P - TCGCGGAGTGTGAAAGGCAGA	
IL-15_onmy	Interleukin 15	Innate immunity	F - TTGGATTTTGCCCTAACTGC	1.09	Raida *et al*. 2011
			R - CTGCGCTCCAATAAACGAAT	
			P - CGAACAACGCTGATGACAGGTTTTT	
IL-8	Interleukin 8	Innate immunity	F - GAGCGGTCAGGAGATTTGTC	1.04	Ingerslev *et al*. 2009
			R - TTGGCCAGCATCTTCTCAAT	
			P - TGTCAGCGCTCCGTGGGT	
MHCI_sasa1	Major histocompatibility complex I	Adaptive immunity	F - GCGACAGGTTTCTACCCCAGT	1.16	Ingerslev *et al*. 2009
			R - TGTCAGGTGGGAGCTTTTCTG	
			P - TGGTGTCCTGGCAGAAAGACGG	
MHCII-B_onmy	Major histocompatibility complex IIβ	Adaptive immunity	F - TGCCATGCTGATGTGCAG	1.01	Raida and Buchmann 2008
			R - GTCCCTCAGCCAGGTCACT	
			P - CGCCTATGACTTCTACCCCAAACAAAT	
MMP13_SASA	Matrix metallopeptidase 13	Multi-function immunity	F - GCCAGCGGAGCAGGAA	1	Tadiso *et al*. 2011
			R - AGTCACCTGGAGGCCAAAGA	
			P - TCAGCGAGATGCAAAG	
NKA_a1b	Na/K ATPase α-1b (saltwater)	Osmoregulation	F - GCCTGGTGAAGAATCTTGAAGCT	0.99	Richards *et al*. 2003
			R - GAGTCAGGGTTCCGGTCTTG	
			P - CCTCCACCATTTGCTCA	
SHOP21	Salmon hyperosmotic protein 21	Osmoregulation	F - GCGGTAGTGGAGTCAGTTGGA	0.92	In house
			R - GCTGCTGACGTCTCACATCAC	
			P - CCTGTTGATGCTCAAGG	
TF_onmy	Transferrin	Stress	F - TTCACTGCTGGAAAATGTGG	0.94	Raida and Buchmann 2009
			R - GCTGCACTGAACTGCATCAT	
			P - TGGTCCCTGTCATGGTGGAGCA	
C7	Complement factor	Innate immunity	F - ACCTCTGTCCAGCTCTGTGTC	1.02	In house
			R - GATGCTGACCACATCAAACTGC	
			P - AACTACCAGACAGTGCTG	
HSC70	Heat shock cognate 70	Stress	F - GGGTCACACAGAAGCCAAAAG	0.96	In house
			R - GCGCTCTATAGCGTTGATTGGT	
			P - AGACCAAGCCTAAACTA	
COIL-P84–2	Coiled-coil domain-containing protein 84	Reference	F - GCTCATTTGAGGAGAAGGAGGATG	1.09	In house
			R - CTGGCGATGCTGTTCCTGAG	
			P - TTATCAAGCAGCAAGCC	
r78d16.1	S100 calcium-binding protein	Reference	F - GTCAAGACTGGAGGCTCAGAG	0.98	In house
			R - GATCAAGCCCCAGAAGTGTTTG	
			P - AAGGTGATTCCCTCGCCGTCCGA	

**Table 3:** Characteristics for each experimental group of tagged coho salmon

Treatment	*N*	Migratory fate				Sex		FL ± SD	Cortisol (ng/mL)		Lactate	Glucose	Sodium
		H	IM	ERM	SUC	f	m	cm	f	m	mmol/L		
C	50	3	7	9	31	-	-	58.2 ± 3.10	-	-	-	-	-
GN	52	0	19	16	31	25	23	59.1 ± 4.13	387 ± 149	138 ± 52.7	7.18 ± 1.79	8.02 ± 2.97	158 ± 12.1
AA	48	5	13	11	19	25	16	59.2 ± 3.75	408 ± 162	138 ± 48.5	7.52 ± 1.45	6.94 ± 1.98	160 ± 15.3
A	50	2	16	14	18	20	23	59.1 ± 4.54	408 ± 84.4	193 ± 146	7.38 ± 1.71	7.85 ± 3.95	158 ± 12.1

For treatment groups, C = control, GN = gill net, AA = angled with air exposure and A = angled with no air exposure. For migratory fate, H = harvested during migration by anglers, IM = immediate mortality, ERM = en route mortality, SUC = successful migrant. Plasma ions and sex steroids were quantified from blood samples taken after each experimental fisheries treatment and not taken in the control group

#### RNA extraction, normalization and cDNA PCR

All samples were processed as per methods outlined in Miller *et al*. (2016) and Teffer *et al*. (2017). In brief, RNA extraction was completed using mechanical abrasion in a MM301 mixer mill (Retsch Inc., Newtown, PA) and TRI Reagent™ followed by addition of 1-bromo-3-chloropropane and purified using MagMAX™-96 Microarray Kits on a Biomek FX^P^ automated liquid handler. Purified RNA quantity and quality were assessed using a Beckman Coulter DTX 880 Multimode Detector (Brea, CA, USA), and sample RNA concentrations were normalized to 62.5 ng/μL. RNA was then converted to cDNA using SuperScript® VILO™ cDNA synthesis kit (Life Technologies) following the manufacturer’s instructions. Specific targeted amplification of target transcripts was performed using primer pairs corresponding to all assays using 1× TaqMan Pre-amp MasterMix as per manufacturer’s instructions (Applied Biosystems, CA, USA). Unincorporated primers were removed using ExoSAP-IT™ (Affymetrix, Santa Clara, CA, USA), and the sample was diluted 1:5 with DNA suspension buffer. The effect of this pre-amplification step on final quantification has been investigated thoroughly and was not found to negatively influence the reliability of results (for extensive detail, see Miller *et al*. 2016). The resulting sample material and assays were loaded directly on to a Fluidigm 96.96 Dynamic Array ™ integrated fluidic circuit chip for qPCR. Combined serial dilutions of artificial construct controls with known copy number were added to the Dynamic Array last and used to track efficiency of each assay on each run. These controls contained an extra probe to track potential contamination (see Miller *et al*. 2016). A series of negative processing controls for RNA extraction, cDNA synthesis and pre-amplification were also included, and a pooled positive control sample of all samples was used in the study.

For host biomarkers, cycle threshold (Ct) is reported for the average of each duplicate biomarker assay using relative expression in the form of 2^−ΔΔCt^ using the averaged expression of two housekeeping genes and the Ct value of a pooled positive control (Livak and Schmittgen 2001). Infective agents were only considered detected if their Ct was below the assay-specific limit of detection (95% level) described in Miller *et al*. (2016) and detected in both duplicate samples. The mean Ct of detected infective agents was calculated and is presented herein as relative load by subtracting the observed Ct value from the total PCR cycles for each qPCR run (i.e. 40-Ct).

### Statistical analysis

All data filtering and statistical tests were conducted using R Statistical Software (R Core Team, 2018). Downloaded telemetry data was filtered, and false detections were removed from fixed receiver stations for any detection that did not (i) correspond with deployed tag’s unique frequency and code designations or (ii) follow 4-s signal transmission intervals. A positive detection was determined by at least two detections within a 20-s period that were multiples of 4 s. All fish taken from the system by anglers were removed from the dataset at the reported time of capture. Post-release behaviour was determined by assessing delay in migration initiation, designated by passage of the first up-river receiver, and swimming ground speed (km/h) between stationary receivers located in Chilliwack River. Pearson’s chi-squared test was used to assess the relationships between fisheries treatment and migratory fate for all tagged fish (i.e. non-biopsied control and treatment groups; *n* = 190).

#### Microbe prevalence, relative load and relative infection burden

Prevalence for each infectious agent detected was calculated for the tracked fish and again for all fish sampled. Pathogens with high prevalence (>70%) within the sample population are analyzed independently using non-parametric Wilcoxon rank sum approximation to assess sex-specific and temporal differences in relative load. To assess the potential effects of high loads within the sample population, infection with rare pathogens and potential cumulative effects of multiple infections, an index accounting for both load and diversity of agents detected within each individual was used to include potential effects of pathogens found in low prevalence. Infectious agents found in each host were summarized as a single variable representing the ‘Relative Infection Burden’ (Bass *et al*. 2019), whereby high loads and both common and rare pathogens contribute to the burden index as follows:

$$ \mathrm{RIB}={\sum}_{i\epsilon m}^m\frac{L_i}{L\max_i}, $$

where *L_i_* is the pathogen relative load (40-Ct) for that sample, *L*max*_i_* is the highest relative load of that agent observed in the sample population and *m* is the sum of *L_i_*/*L*max*_i_* for all pathogens detected in the sample.

The relationship between RIB, sex and treatment on blood physiology was tested using multivariate analysis of variance (MANOVA) with Type I sum of squares to account for sex-specific variation in physiological variables. Ordinal logistic regression models (*polr* function in MASS r package), as well as GLMs (family = binomial, link = logit) with binary response (survival to spawning habitat) were used to investigate the influence of RIB, treatment and sex on migratory fate and survival. RIB, blood ions and metabolites were log_10_-transformed to address non-normal distributions for regression models; otherwise, non-parametric tests were used in cases where data structure was in violation of parametric test assumptions.

### Transcription profiles and infectious agent communities

Principal component analysis was used to reduce dimensionality of gene expression data. Scree plots were used to visually estimate the number of components to retain (cumulative variance > 50%), and Bartlett’s test of sphericity was used to ensure dataset structure was suitable for ordination. Factors influencing gene expression principle components (PCs) were investigated using generalized linear models with PCs as the dependent variable. Model covariates included RIB, blood ions (i.e. potassium, sodium), metabolites (i.e. glucose, lactate), cortisol, sex and survival.

Infectious agent community composition was summarized using non-metric multidimensional scaling (NMDS) ordination with a Bray–Curtis dissimilarity metric, with each host as an individual replicate. NMDS is well suited for community data because NMDS is not restricted by linear ordination structures as it uses non-Euclidean distance measures in *n* dimensional space and can thus accommodate non-parametric and count data. We used the *metaMDS*, *envfit* and *anosim* functions from the R package *vegan* (version 2.9.2) to create ordinations and investigate the relationship between pathogen community structure and transcription profiles, fate and sampling time. The optimal number of dimensions to include in each ordination was visually assessed using scree plots. In NMDS, the calculated stress statistic represents the level of dissimilarity within the original data captured by the produced ordination, where values < 0.2 are generally accepted as indicating an interpretable representation of data dissimilarity for ecological systems (Kruskal and Wish 1978; Jaworska and Chupetlovska-Anastasova 2009).

## Results

### Fisheries treatments and post-release mortality

Of the 200 tagged and released fish, 42.5% successfully migrated to spawning habitat, 27.5% were immediate post-release mortalities and the remaining 30% were en-route mortalities. A high degree of variation in migratory behaviour was observed, with migration times ranging between 2.9 and 37 days (mean = 14.1 days) to traverse the 65-km from the release site to the hatchery. Six percent (*n* = 12) were reported as captured in fisheries, two of which were captured near spawning habitat and included in analysis. A total of 147 samples were screened for infectious agents and gene expression characterized: 112 gill samples at tagging release, 20 from fish recaptured post migration and 16 baseline samples.

**Figure 2:**
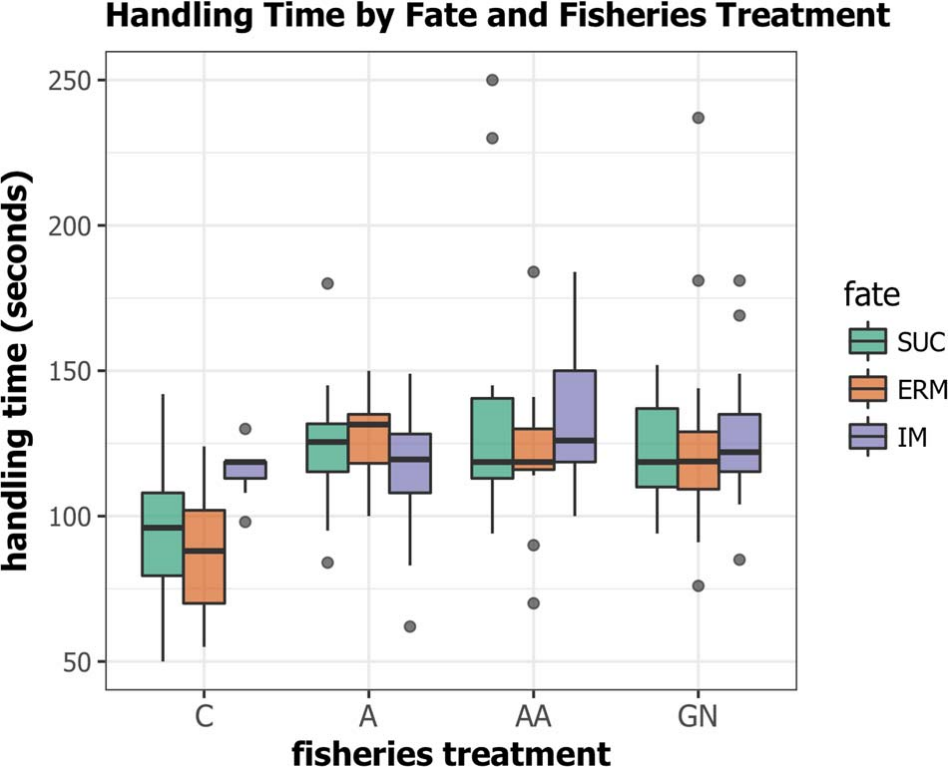
Standardized handling time and migratory fate for coho salmon exposed to experimental fisheries treatments (C: control with no fisheries treatment; A: fish angled for 2 min with no air exposure; AA: fish angled for 2 min and air exposed for 1 min; GN: fish entangled in 140 mm gill net for 3 min and air exposed for 1 min). Handling time excludes experimental treatment duration and air exposure. Migratory fate as IM for immediate mortality, ERM for en route mortality and SUC for successful migration as determined by tracking fish using radiotelemetry post-release

Migratory fate (immediate mortality, en-route mortality or successful migration) was significantly related to treatment group (*χ*^2^ = 13.03, df = 6, *P* = 0.04; Fig. 2); treatment fish experienced somewhat greater immediate post-release mortality compared to non-handled controls (*χ*^2^ = 6.45, df = 3, *P* = 0.07), and control fish were more likely to reach spawning habitat compared to all treatment groups (*χ*^2^ = 12.6, df = 3, *P* = 0.005). Among treatment groups, there was no difference in migratory fate (*χ*^2^ = 1.33, df = 4, *P* = 0.856; Table 3). Plasma cortisol was significantly higher in females than in males (*t* = 10.641, df = 106.57, *P* < 0.001) and was not related to experimental fisheries treatments (*F*_2,89_ = 0.5623, *P* > 0.05), and there was no significant interaction between sex and treatment (*F*_2,89_ = 0.1461, *P* > 0.05). Post-release behaviour characterized by migratory delay, total duration of migration and swim rate through low and high velocity flow portions of the river was not significantly predicted by treatment (two-way MANOVA; *F*_12,213_ = 0.89, *P* = 0.557), handling time (*F*_4,69_ = 1.61, *P* = 0.182) or their interaction (*F*_12,213_ = 0.3, df = 9, *P* = 0.975). There were no significant differences in sex (*χ*^2^ = 3.32, df = 3, *P* = 0.344) or fork length (*t* = 0.88, df = 128, *P* = 0.380) among treatment groups.

Handling time was significantly lower for control fish compared to all treatment groups (Kruskal–Wallis *χ*^2^*P* < 0.001) with no significant variation among other treatments (Kruskal–Wallis *χ*^2^*P* > 0.05; Fig. 2). The difference in handling time between control and fisheries treatments was 24.1 s and represents the time required to take gill biopsies and blood samples. Handling time significantly predicted migratory fate for controls (Kruskal–Wallis *χ*^2^*P* = 0.006; Bonferroni corrected for multiple comparisons *α* = 0.0125) but not for fish exposed to fisheries treatments.

### Factors associated with infection burden

**Figure 3:**
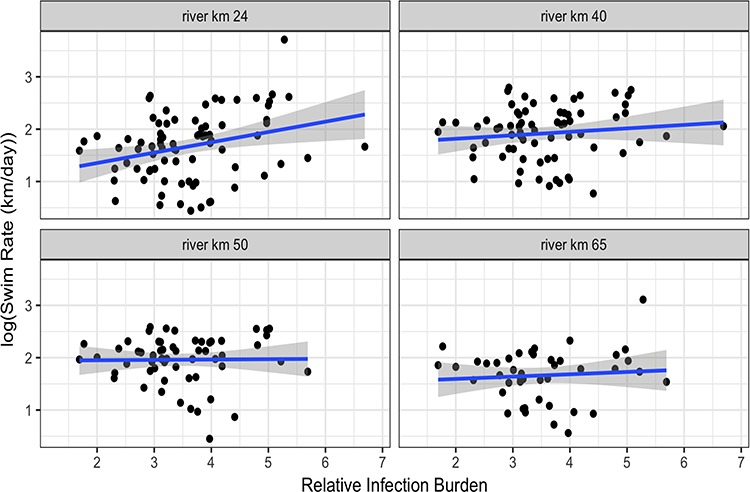
Up-river migration rate and relative infection burden (RIB) of tagged coho from release point and between receivers in the Fraser/Chilliwack River system. A positive relationship between RIB and migration initiation post-release was found; however, this relationship is not maintained for the remainder of the migration

**Figure 4:**
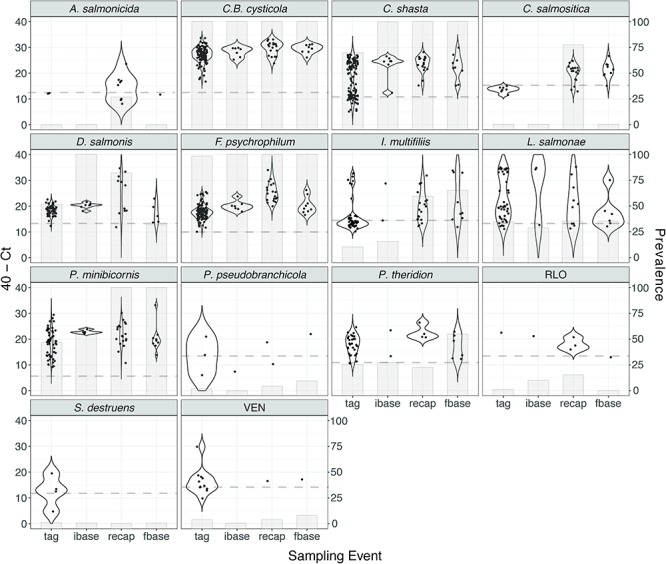
Relative loadings and prevalence of infectious agents detected at each sampling event. Prevalence values do not consider detections that were under the limit of detection (LOD), denoted by the dashed line. Sampling events are abbreviated as follows: samples taken at release (tag), initial baseline (ibase), upon recapture after migration (recap) and final baseline (fbase)

Fourteen infectious agents were detected in the screened population, including four bacteria (*Aeromonas salmonicida*, *Flavobacterium psychrophilum*, ‘*Candidatus* Branchiomonas cysticola’, *Rickettsia*-like organism [RLO]), nine microparasites (myxozoans: *Ceratonova shasta*, *Parvicapsula pseudobranchicola*, *Parvicapsula minibicornis*; protists: *Dermocystidium salmonis*, *Sphaerothecum destruens*; flagellate: *Cryptobia salmositica*; ciliate: *Ichthyophthirius multifiliis*; microsporidea: *Paranucleospora theridion*, *Loma salmonae*) and one virus (viral erythrocytic necrosis [VEN]; Table 1; Fig. 4). All screened gill samples contained at least two infectious agents. Relative loads and prevalence did not vary by sex except for increased prevalence of *L. salmonae* and high relative loads of *C. shasta* in females compared to males (Wilcoxon *χ*^2^ approximation *P* = 0.017). RIB of tagged fish at the time of capture was not significantly different among treatment groups (*F* = 1.87, *P* = 0.160) or between sexes (*F* = 1.7, *P* = 0.195). RIB, treatment, sex and their interaction terms were not predictive of migratory fate (ordinal logistic regression: *P* > 0.05). Post-release migratory delay was weakly related to RIB; fish with higher infection rates initiated migration sooner compared to less infected individuals independent of sex, treatment and blood cortisol values (*R*^2^ = 0.12, *P* = 0.003; Fig. 3). Beyond 24 rkm, however, there was no effect of RIB on migration rate for any river segment or total remaining migration. ANOVA tests on individual blood ions and hormones (predictor variables: sex, RIB, treatment, RIB–sex interaction, RIB–treatment interaction) revealed significant effects of sex and RIB on blood cortisol only, where higher RIB values are associated with lower circulating blood cortisol levels (Table 4).

**Table 4:** ANOVA effect tests for cortisol, blood ions and blood metabolites for sex, experimental fisheries treatments, relative infection burden (RIB) and their interactions with RIB from adult migratory Coho salmon.

Factor	Predictor	df	SS	*F* ratio	*P* value
Cortisol	Sex	1	27.7239	171.7269	**<0.001** ^***^
	RIB	1	1.6279	10.0832	**0.00199** ^**^
	Treatment	2	0.0579	0.1795	0.836
	Treatment^*^RIB	2	0.6469	2.0035	0.140
	Sex^*^RIB	1	0.2763	1.7117	0.194
Glucose	Sex	1	0.0520	0.4743	0.493
	RIB	1	0.1007	0.9194	0.340
	Treatment	2	0.2763	1.2614	0.288
	Treatment^*^RIB	2	0.4726	2.1577	0.121
	Sex^*^RIB	1	0.0002	0.0019	0.965
Lactate	Sex	1	.0514	0.9983	0.320
	RIB	1	.0479	0.9309	0.337
	Treatment	2	.0616	0.5991	0.551
	Treatment^*^RIB	2	.0773	0.7507	0.477
	Sex^*^RIB	1	.0097	0.1879	0.667
Chloride	Sex	1	0.08360	2.7273	0.102
	RIB	1	0.00116	0.0380	0.846
	Treatment	2	0.03076	0.5017	0.607
	Treatment^*^RIB	2	0.00714	0.1164	0.890
	Sex^*^RIB	1	0.00044	0.0143	0.905
Sodium	Sex	1	0.01591	2.4395	0.121
	RIB	1	0.00036	0.0550	0.815
	Treatment	2	0.00699	0.5358	0.589
	Treatment^*^RIB	2	0.00541	0.4145	0.662
	Sex^*^RIB	1	0.02200	3.3732	0.0692
Potassium	Sex	1	0.1587	0.7118	0.401
	RIB	1	0.0596	0.2671	0.606
	Treatment	2	0.9399	2.1078	0.127
	Treatment^*^RIB	2	0.6032	1.3527	0.263
	Sex^*^RIB	1	0.3509	1.5738	0.212

Significance indicated in bold, p <0.0001 (***), p < 0.001 (**), p < .01 (*)

**Figure 5:**
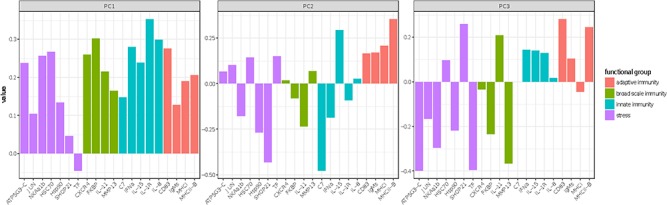
Eigenvalues from retained principal components of biomarker transcripts from migratory adult coho salmon, representing 32.2, 16 and 9% of the variation among the data, respectively.

**Figure 6:**
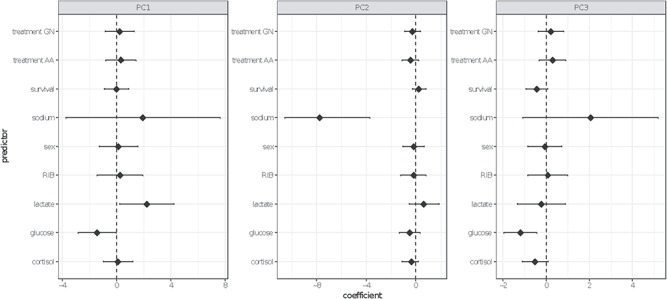
Forest plot of predictor coefficients from generalized linear models for transcriptional profiles of adult migratory Coho salmon represented by principal component analysis. Factors are abbreviated as follows: GN: gillnet; AA: angling with 1 min air exposure; RIB: relative infection burden

**Figure 7:**
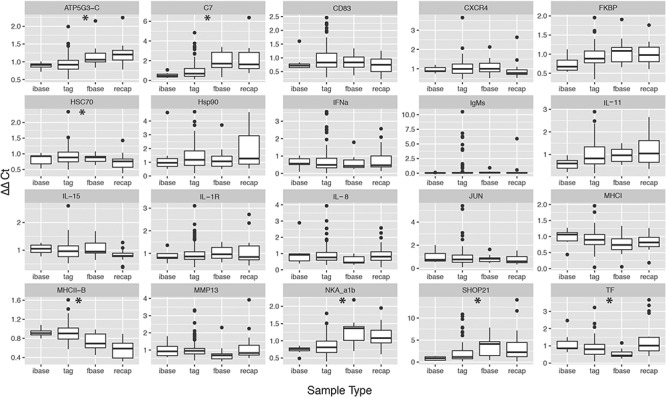
Gene expression of biomarkers associated with stress and immune function from migratory adult coho salmon collected from the Chilliwack hatchery in the Fraser River watershed. Axis labels represent type of sample: ibase (initial baseline sample *n* = 7), tag (sample from experimentally relocated and fisheries treated fish *n* = 112), recap (fish recaptured post-migration; *n* = 19) and fbase (final baseline sample *n* = 9). Asterisks represent significance based on Kruskal–Wallis ranked sum with Bonferroni correction for multiple comparison at *P* = 0.0025

**Figure 8:**
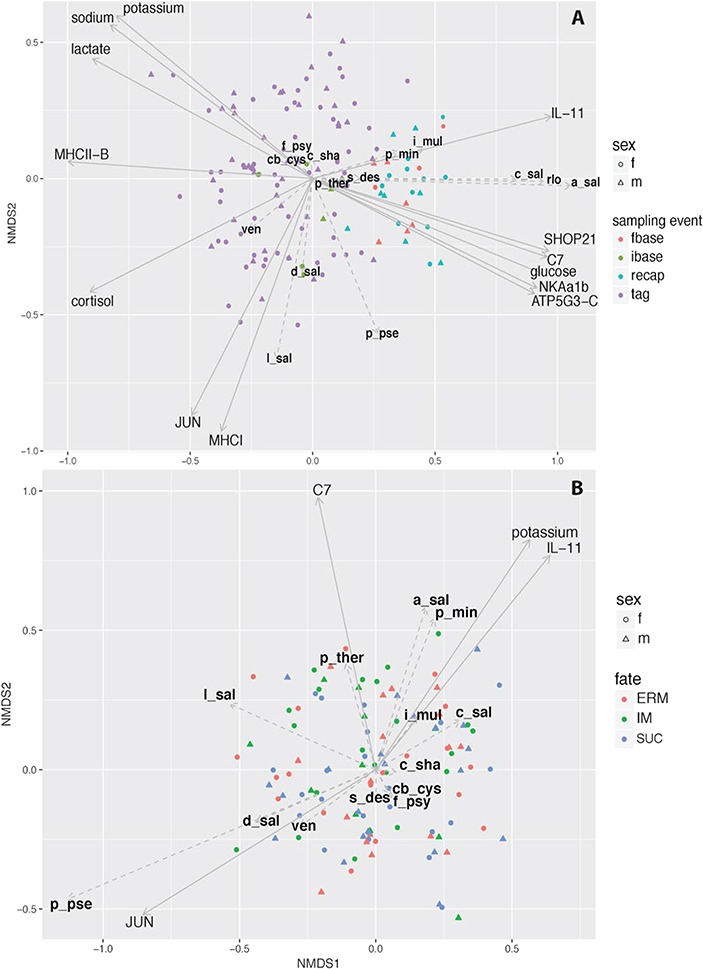
NMDS ordinations of infectious agent relative load for (**A**) all samples, excluding initial samples from recaptured individuals and (**B**) samples at tagging. Arrows represent biomarkers and blood parameters significantly correlated with the ordination (*P* < 0.05). Ordination A was significantly related to the time of sampling (sample type: ibase = initial baseline, fbase = final baseline, tag = at release, recap = at recapture post-migration). Migratory fate (ERM = en route mortality, IM = immediate mortality, SUC = successful migration) was not significantly related to Ordination B.

Increases in RIB due to higher prevalence and loads of several pathogens were observed in recaptured fish sampled post-migration, supporting pathogen accruement during migration (Fig. 3). Comparing initial and final samples, *A. salmonicida*, *C. salmositica* and RLO were not detected at release but were detected upon recapture, and significant increases in the relative loads of Ca*.* B*. cysticola* (*P* = 0.044), *C. shasta* (*P* = 0.0274), *F. psychrophilum* (*P* < 0.001) and *I. multifiliis* (*P* = 0.004) were observed*.* Relative loads of detected infectious agents were similar in recaptured and final baseline groups except for higher loads of *F. psychrophilum* in fish that completed the migration twice (Wilcoxon ranked sum *P* = 0.001).

### Transcriptional profiles

Three retained PCs summarized 57.2% of the ordination variation (Fig. 5). No single genes or functional gene groups contributed maximally to PC1. Stress-associated genes HSP90 and SHOP-21 and innate immune gene C7 loaded negatively on PC2, and adaptive immune genes MHCI and MHCII-B and cytokine IL-15 loaded positively on PC2. Genes associated with stress ATP5G3, TF and SHOP 21, as well as broad-scale immunity and inflammation MMP13, were maximal contributors to PC3. Fish that were recaptured showed distinct separation in PC structures, particularly in PC2 (Fig. 5). Generalized linear models to investigate factors influencing transcriptional profiles of tracked coho salmon identified effects of blood ions and metabolites, but no significant contribution of sex, treatment, RIB or migratory fate (Fig. 6). Blood lactate and glucose are significant covariates explaining PC1, (*P* = 0.03, *P* = 0.04 respectively), while blood sodium levels alone were highly predictive of gene expression summarized in PC2, summarizing osmoregulatory, immune and stress genes associated with maturation (*P* < 0.001). PC3, representing stress and broad scale immune genes, was strongly associated with blood glucose (*P* = 0.002) and weakly associated with blood cortisol values and migratory success (*P* = 0.09).

No patterns in gene expression associated with migratory fate were observed. Transcription of several genes differed when comparing individuals sampled upon recapture (Fig. 7); significant up-regulation in ATP5G3-C, C7, SHOP 21, TF and NKA a1b was observed in fish recaptured post migration (Kruskal–Wallis ranked sum *P* < 0.007), and notable downregulation in HSC70 and MHCII-B (*P* < 0.01). Small sample size of initial and final baseline samples and high variance in samples taken upon tagging prohibits interpretation of tagging effects on gene expression for individual genes.

### Gene expression, infectious agent community and fish condition

NMDS ordination of infectious agent community yielded a stress value of 15.8, indicating a good level of dissimilarity represented by the ordination. ANOSIM analysis of sample type (initial and final baseline, tagging and recapture) revealed changes in infectious agent community structure over time (*P* < 0.001, ANOSIM statistic *R* = 0.2; Fig. 8A). Gene expression summarized by PC2 was highly associated with infectious agent community structure based on *envfit* analysis (*r*^2^ = 0.17, *P* < 0.0001). NMDS of only samples taken at tagging produced a similar stress value of 16.0. ANOSIM analysis revealed no significant relationship between migratory fate and infectious agent community (*P* = 0.532; Fig. 8B). *Envfit* analysis of NMDS ordinations with transcription profile PCs was significant but with small effect size for PC3 (*r*^2^ = 0.03, *P* = 0.03). More detailed analysis of the infectious agent community ordination using individual biomarkers and physiological variables revealed no distinct signal when considering only tagged fish; however, a strong osmoregulatory response is associated with species that increase in prevalence and relative load in fish sampled at the end of the study (i.e. final baselines and recaptured fish).

## Discussion

This study sought to characterize factors associated with coho salmon health and condition to investigate the incidence and mechanisms of potential capture-mediated pathogen-induced mortality in wild salmon following fisheries interactions. All infectious agents detected are considered endemic and within prevalence ranges observed in previous surveys in the southern BC region (Miller *et al*. 2014; Bass *et al*. 2017; Teffer *et al*. 2018; Table 1). Fisheries treatments were associated with decreased survival compared to controls, and handling time alone was related to mortality of control fish, with longer handling times associated with increased post-release mortality regardless of treatment. As there was no effect of fisheries treatment on measured physiological variables, variation in individual condition regardless of fisheries treatment was investigated. Physiological parameters were significantly associated with gene expression and sex but not with migratory behaviour and survival. We identified a negative correlation between cortisol and infectious agent burden that was sex-specific but not predictive of survival. Finally, infectious agent community composition was predictive of immediate post-release migratory behaviour but not migratory fate, and changes in infectious agent community and salmon physiology were observed over the course of the study and were associated with biomarkers related to osmoregulation, metabolism and stress.

### Fisheries stress and post release survival

The combined post-release mortality of 57.5% (27.5% immediate, 30% delayed) observed in this study is high compared to previous estimates for adult coho (Raby *et al*. 2014, 2015a) and likely a result of cumulative effects of the repeated migration and extending post-release monitoring duration. Although non-biopsied controls survived better than treated fish, impairment related to the severity of fisheries treatment was not observed; post-release migratory behaviour and success were similar among fisheries treatments. Our results are contrary to previous research on Pacific salmon, where experimental gill-net entanglement was related to elevated indicators of stress physiology and migratory delay post-release compared to control groups (Nguyen *et al.* 2014; Keefer *et al*. 2017; Teffer *et al*. 2017; Bass *et al.* 2018). Previous work has demonstrated high resilience to fisheries stressors as salmon approach spawning grounds (Raby *et al*. 2013; Bass *et al.* 2018). Coho salmon experimentally treated with fisheries stressors at temperatures similar to those in this study (10.7–12.5°C) were resilient to capture stress, while fish exposed to high temperature (15°C) experienced significantly higher mortality (Teffer *et al*. 2019). Consequently, coho included in this study may have been resilient to the variation in severity of fisheries treatment, yet vulnerable to the energetic and physiological demands associated with transport and repetition of 75 km of migration experienced during this study.

The amount of time a fish was handled, regardless of treatment group, was found to influence immediate mortality post-release. Raby *et al*. (2012) found no relationship between fish handling time and migratory fate in wild coho salmon released from beach seine fisheries, contrary to marginally non-significant results of the present study. Harmful factors unique to handling include loss of mucous coat and externally applied pressure (reviewed in Brownscombe *et al.* 2017; Cook *et al.* 2019), which are influenced by the duration of handling. Research assessing the impacts of various tagging methods on physiological indicators of sockeye salmon found capture, handling and holding effects were more pronounced than tagging alone (Dick *et al.* 2018). In this study, all fish handlers had extensive experience working with adult salmon and the biopsy and tagging methods employed have been widely applied with high survival rates (Donaldson *et al*. 2012; Nguyen *et al.* 2014; Raby *et al*. 2013; Bass *et al.* 2018; Bass *et al*. 2019). Variation in handling times was attributed to a diversity of factors and was not defined by gear type but rather practical constraints; for example, handlers often need to pause or use additional restraint while a fish is struggling to prevent its escape. This is particularly true for control fish that were not exposed to exhaustive exercise prior to handling, as they are more vigorous during tagging. While not explicitly recorded as part of the experimental design, long handling times of control fish are likely attributed to such cases despite best efforts to minimize handling stress.

The significant negative effect of handling alone on fish survival independent of fishery treatment is a critical consideration for best practices in catch-and-release fisheries. Many best-practice recommendations prioritize reducing air exposure over handling time (e.g. Brownscombe *et al*. 2017). Yet, our results indicate that the duration of handling time alone can have a greater impact on post-release survival in some fisheries. In sectors where catch-and-release is used as a conservation strategy by managers, best-practice recommendations should stress the importance of reducing the duration of all interactions with captured fish alongside gear and air exposure recommendations.

### Relative infection burden and sex-related stress response

Consistent with previous research on migratory Pacific salmon in the region (e.g. Bass *et al*. 2017; Teffer *et al*. 2019), RIB increased in fish resampled post-migration. Condition of fish upon recapture was markedly different from condition at tagging; recaptured fish demonstrated advanced sexual dimorphism, spawning preparedness, scale reabsorption and development of visible fungal infections. Natural recruitment of infectious agents occurs during spawning migrations, particularly at spawning grounds when fish are in high densities and immunocompromised (Miller *et al*. 2014; Teffer *et al*. 2019). The significant increase in prevalence and relative loads of *C. salmositica* in recaptured fish suggests that fish taken from the hatchery and transported downstream had not been in fresh water for an extended period of time; previous work at the same hatchery noted high prevalence and relative loads of *C. salmositica* with extended residency in hatchery channels (Bass *et al.* 2018). The load and prevalence increase of freshwater bacteria (*F. psychrophilum*) and microparasites (*I. multifiliis*) also support temporal increases that occur during migration (Teffer *et al*. 2017; Bass *et al.* 2018). Higher prevalence and loading of *F. psychrophilum* in recaptured fish compared to those sampled at the hatchery at the end of the study (final baseline samples) may indicate the effects of experimental relocation, treatment, and handling on resistance to *F. psychrophilum* infection. Considering *F. psychrophilum* has been identified as an infectious agent that poses moderate risk to Pacific salmon (Miller *et al*. 2014), potential increase in vulnerability to infection as a result of fisheries stress and handling warrants further investigation.

There was no observed interaction between fisheries treatments and RIB and neither of these factors predicted migratory fate. The fact that high RIB was associated with shorter migratory delay suggests that high relative loads of multiple infectious agents are related to intensified up-river migration behaviour. Infection rates have been demonstrated to increase as Pacific salmon get closer to spawning (Bass *et al.* 2018; Teffer *et al*. 2019); available energy is depleted, and immune response is significantly diminished (Dolan *et al*. 2016). The semelparous life history strategy seen in Pacific salmon has selected for reallocation of energy away from physiological processes not directly associated with gamete production and spawning behaviour such as immune function (Teffer *et al*. 2019); consequently, individuals with higher RIB may be further along their senescence trajectory compared to those with lower RIB (Dolan *et al*. 2016; Teffer *et al.* 2019). In contrast, previous work found that pathogens such as *C. salmositica* is associated with decreased migration rates (Bass *et al.* 2018). Here, the prevalence of *C. salmositica* in the tagged sample population was too low to detect an effect. Accelerated migration rates associated with fish condition similar to those observed here have been observed in compromised Sockeye salmon (Drenner *et al.* 2018; Miller *et al*. 2011).

Interestingly, RIB was negatively related to circulating cortisol levels in this study, and this relationship was more pronounced for males than for females. Previous research on adult coho salmon has identified sex-specific differences in response to stressors, including prolonged physiological disturbance after gill net entanglement in females compared to males (Teffer *et al*. 2019). Female Pacific salmon have been consistently shown to have higher circulating blood cortisol levels compared to males (Kubokawa *et al*. 2001; Cook *et al*. 2011; Raby *et al*. 2013) independent of infection burden (Teffer *et al*. 2019). Additionally, glucose metabolism is higher in females during spawning migrations, indicating sex-specific energy demands and metabolism (Teffer *et al*. 2017, 2019). The lack of observed correlation between RIB and cortisol in females may be attributed to such sex-specific energy allocation and stress responsiveness. The negative association of RIB with circulating cortisol in males may indicate an impaired or suppressed stress response associated with high infectious agent burden or a more advanced stage of senescence. This result differs from previous work on Chinook salmon released after mild gill-net entanglement, where no relationship between RIB and circulating cortisol for males or females was detected (Bass *et al*. 2019). However, migratory sockeye salmon *O. nerka* with visual pathologies associated with fungal infections (likely *Saprolegnia* ssp.) had elevated cortisol levels compared to those without visible infection both in-river and at spawning grounds (Baker *et al*. 2013). These results indicate that further research is necessary regarding potential impaired stress response in Pacific salmon associated with infective agent burden.

### Transcription profiles

The lack of detectable relationship between transcription profiles, RIB and migratory fate highlights the challenges associated with characterizing individual health and post-release survival outcomes in wild fish. Gene transcription relating to acute stressors can be highly transient and variable. Alternately, chronic stressors responses are more stable and consistent; in fact, recent research has shown that fish experiencing chronic thermal or osmotic stress can be specifically identified based on the co-activation of panels of 8–10 genes (Houde *et al*. 2019). Unfortunately, we did not have such panels at the time of this study and rather employed a broad range of genes known to be activated when fish are experiencing stress or responding to infection. While these can provide clues as to the range of physiological processes being activated, especially when combining with other measured variables such as blood chemistry and infectious agents, they are not as informative of more curated panels. Given the high variability in exposure of free-ranging salmon to stress, it may be necessary to employ larger sample sizes and attempts to reduce confounding effects (e.g. transport stress) to detect transcriptional signals that have been observed in more controlled experimental laboratory holding studies (e.g. Teffer *et al*. 2017, 2019).

In our study, gene transcription was, however, related to aspects of individual physiology; plasma ions and metabolites were significantly related to transcription profiles summarized by PC1 and PC2 at release, yet there was no detected secondary stress response to experimental fisheries treatments. Donaldson *et al*. (2012) found that changes in transcription of stress associated genes peaked 2–4 h post induction of an acute stressor, while lactate and cortisol took ~24 h to return to baseline levels. Here, sampling occurred 2–5 h after initial capture. Transcriptomic response to transport and holding stress is not easily observed in this study given the small sample sizes for initial baseline and high variance in individual gene transcription. However, given that lactate and glucose are known biomarkers of exhaustive exercise (Barton 2002), and sodium and glucose together have been associated with stress response in salmon (Wagner and Congleton 2004), the gene expression profiles represented by PC1 and PC2 may describe broad-scale transcriptional response associated with the exercise and stress experienced during initial capture and transport.

Several biomarkers of a stress and immune response that have previously been associated with mortality in salmonids (SHOP21, ATP5G3, TF, MMP13) and several immune genes (CD83, MHCII-B, MMP13) were major contributors to PC3, which was related to plasma glucose and marginally associated with cortisol and migration survival in tracked fish. When considering changes over time, all stress-related genes that contributed maximally to PC3 demonstrated a significant fold increase in transcription post-migration, while MHCII-B expression decreased significantly. These patterns are consistent with senescence in Pacific salmon, including adult coho (Teffer *et al*. 2019) and suggest that transcriptional profiles represented by PC3 may be associated with senescence and/or spawning preparedness. Stress-associated genes were also linked with the infectious agent community composition of tagged coho at release and recapture; however, the low variation explained by PC3 and small effect size of the relationship indicates a highly variable physiological (Cooke *et al*. 2006) and/or transcriptional (Hammill *et al*. 2012; Teffer *et al*. 2019) response to stress. Indeed, although blood glucose is typically positively related to cortisol levels, semelparous salmonids sampled during spawning migrations have demonstrated variable and muted glucose responses relative to cortisol levels (McConnachie *et al*. 2012; Dick *et al.* 2018).

The relationship between infectious agent communities and gene expression when considering changes over time highlights stress and immune genes that have previously been associated with both increased infectious agent loads and senescence (PC2 SHOP21, C7 and MHCIIB; Teffer *et al*. 2017). Previous experimental work on sockeye salmon observed similar trends: individuals with high loads of *F. psychrophilum* and *C. shasta* demonstrated similar expression profiles of stress and immune genes including SHOP21 and C7, while MHCIIB was associated with *P. minibicornis* and *Ca.* B. cysticola (Teffer *et al*. 2017). As semelparous fish undergo senescence and infection burdens increases, osmotic stress also increases. Damage to the integument can be caused by agents such as *F. psychrophilum* and *Ca*. B. cysticola, which have also been associated with osmotic impairment and subsequent mortality (Miller *et al*. 2011; Jeffries *et al*. 2012). The observed up-regulation of SHOP21 and C7 post-migration corresponding with downregulation of MHCII-B may be in response to disease associated osmotic stress resulting from increased infection burden and decreased immunocompetency. Together, these results indicate the synergistic trajectory of natural senescence and disease ecology in Pacific salmon and highlight the challenges associated with teasing apart causal relationships among gene expression, stress physiology and the numerous infectious agents carried by wild salmon in the final stages of freshwater migration. However, as these methods are applied across multiple species, populations and freshwater ecosystems, the similarities in connections and relationships observed will inform associations that may indeed be causal.

The experimental manipulation of fish that includes downstream transport is not entirely realistic (Cooke *et al*. 2013); however, such approaches facilitate experimental comparisons and enable the application of mechanistic and comparative approaches to understanding the relative effects of different fisheries stressors to inform evidence-based conservation (Cooke *et al*. 2017). Investigating fisheries stress responses independent of capture or confinement effects is a logistical challenge for stress physiology research in wild animals (Patterson *et al*. 2016). Previous work on Pacific salmon has shown even temporarily holding actively migrating fish is itself stressful with potential to result in near total mortality upon releasing fish (Donaldson *et al*. 2011). Therefore, experimental effects cannot be easily addressed with changes in blood sampling time. Further, collecting fish that had already reached the hatchery biased our sample population towards surviving fish, which may carry lower infectious agent loads than those that died en-route. To further understand the context-specific responses of fish to fisheries capture, intercepting fish during migration enhances the likelihood of detecting highly pathogenic infectious agents or fish in poor condition. Due to naturally occurring co-infections (every fish sampled was positive for at least two infectious agents), co-infection dynamics should also be considered by future studies. This method would further benefit the investigation of co-infection dynamics and sex-specific responses to infection and fisheries stress in the wild; however, large sample sizes are required to account for pathogens occurring in low prevalence.

Several factors may have influenced our ability to detect disease-related transcriptional response and mortality in this study. While the panel of infectious agents used here includes most pathogens likely to be present on the Pacific coast, there is potential for unknown agents to exist within the host sample population. Pathogens not detected by our methods may decrease our ability to characterize transcriptional profiles of infected versus uninfected fish because presumed healthy fish may indeed be inducing associated immune responses (Miller *et al*. 2017). Any disease-induced mortality associated with undetected pathogens remains unknown. Finally, non-lethal sampling necessitates non-invasive collection of gill tissue and while this method has been demonstrated to have high concordance with pooled multitissue sample (Teffer *et al*. 2019), there is potential for over-representation of agents infecting the gill (*Ca*. B. cysticola and *L. salmonae*) and under-representation of pathogenic agents found commonly in other tissues (e.g. *P. minibicornis*, *T. bryosalmonae*).

## Conclusions

Characterizing the factors associated with individual variation in the susceptibility of Pacific salmon to post-release mortality is a complex challenge that requires the consideration of both external and internal environments at time of capture. Recent evidence suggests a role of infectious agents in mediating post-release survival (Bass *et al*. 2017; Patterson *et al*. 2017; Teffer *et al*. 2017), and our results support an influence of cumulative infection profiles on migratory delay following capture and release. We identified a sex-specific link between RIB and the stress response, with stronger relationships among males. A broad-scale response in transcription of stress and immune genes may have been reflective of capture, transport and confinement that was reflected in physiological variables measured from the blood.

The degree to which individual infectious agents influence the fate of salmon released from fisheries deserves additional and long-term investigation due to the challenges associated with the low prevalence of highly pathogenic species, co-infection dynamics and individual and temporal variation in host immunocompetence. Further, as variability in river temperatures and flow increases with climate change, having a solid foundation of knowledge is critical to adequately monitor the effects dynamic environmental conditions and potential introduction of new infectious agents to Pacific waters (Fenkes *et al*. 2016). Our findings increase our understanding of naturally occurring infections in wild Pacific salmonids and how these infectious agents increase over the course of migration alongside changes in immune function and stress physiology, information that is critical for ongoing monitoring efforts and research as environmental conditions change. Additional insight regarding context-specific response to fisheries interactions is necessary (Raby *et al.* 2015b). Further research on wild salmon is required to identify specific infectious agents and transcription profiles that are predictive of negative outcomes in migrating coho salmon in the wild.

## Funding

This work was supported by the Ocean Tracking Network. S.G.H. and K.M.M were supported by a Natural Science and Engineering Research Council of Canada (NSERC) Strategic grant and funding from Genome BC. S.J.C. is a Canada Research Chair and supported by an NSERC Discovery Grant. J. Chapman received support from the Natural Sciences and Engineering Research Council of Canada.

## Supplementary Material

Suppl_data_coaa033
